# From the Wild to the Field: Documentation, Propagation, Pilot Cultivation, Fertilization, and Phytochemical Evaluation of the Neglected and Underutilized *Amelanchier ovalis* Medik. (Rosaceae)

**DOI:** 10.3390/plants12051142

**Published:** 2023-03-02

**Authors:** Eleftherios Karapatzak, Theodora Papagrigoriou, Katerina Papanastasi, Olga Dichala, Antonis Karydas, Nikos Nikisianis, Giorgos Patakioutas, Diamanto Lazari, Nikos Krigas, Eleni Maloupa

**Affiliations:** 1 Institute of Plant Breeding and Genetic Resources, Hellenic Agricultural Organization Demeter, 57001 Thessaloniki, Greece; 2 Laboratory of Pharmacognosy, School of Pharmacy, Aristotle University of Thessaloniki, 54124 Thessaloniki, Greece; 3 Systems of Forest and Environmental Development (SYSTADA), 8 Amasia, 55133 Thessaloniki, Greece; 4 Department of Agriculture, School of Agriculture, University of Ioannina (UOI), 47100 Ioannina, Greece

**Keywords:** snowy mespilus, serviceberry, native germplasm resources, superfood fruit crops, asexual propagation, ex situ conservation, nutrient management

## Abstract

The snowy Mespilus, or serviceberry (*Amelanchier ovalis* Medik., Rosaceae) represents a neglected and underutilized small fruit tree species with high nutritional value. In this work, we present the results of a long-term study facilitating the sustainable exploitation of *A. ovalis* as a new germplasm resource from the Greek flora. Ten wild-growing population samples of *A. ovalis* have been collected from natural habitats in northern Greece. Asexual propagation trials on these materials delivered successful propagation (83.3% rooting) on a selected genotype via leafy cuttings of young, primary, non-lignified soft wood with the application of the rooting hormone. The ex situ cultivation potential of the selected genotype has been evaluated under distinct fertilization regimes in a pilot field trial. Three-year results of this ongoing trial have shown that *A. ovalis* does not require external nutrient enhancement to be established during its early stages since plant growth rates between conventional fertilization and control plants were similar for the first two years and higher compared to organic fertilization. Conventional fertilization delivered higher fresh fruit production in the third year, with higher fruit number and fruit size compared to organic fertilization and control plants. The phytochemical potential of the cultivated genotype was assessed via the total phenolic content and radical scavenging activity of separate extracts from leaves, twigs, flowers, and young fruits, which revealed that individual plant organs have strong antioxidant activity despite their moderate total phenolic content. The multifaceted approach applied herein has provided novel data that may set the framework for further applied research toward the sustainable agronomic exploitation of Greek *A. ovalis* as a diversified superfood crop.

## 1. Introduction

In the context of sustainable agronomic exploitation, the native, neglected, and underutilized germplasm resources of different regions can convey significant potential as sources of genetic material [1]. A wide variety of plant species with significant agro-alimentary potential can be found in several countries’ native germplasm pools, and relevant research is ever-increasing to date [1,2,3,4,5,6,7,8], particularly on halophytic species [9]. In addition, the potential for strategic utilization of native germplasm resources has recently been brought forward for several ornamental, medicinal, and nutraceutical species across the Mediterranean basin and should also be extended to poorly documented rare and locally endemic species [1,6,7,10]. Native tree species of different regions in particular can produce fruits of high nutraceutical value as a natural source of antioxidants. These antioxidants have the potential to convey significant health benefits and thus can be characterized as superfoods upon consumption [8,11,12]. Greece is a well-known biodiversity hotspot in the context of Europe and the Mediterranean region [6]. Currently, coordinated efforts are underway to identify, select, domesticate, and utilize neglected native plant germplasm of potentially high agronomical value based on its nutraceutical potential through a multifaceted evaluation framework [6,8,13,14].

The acquisition of data that can set the groundwork for exploring the utilization potential of native germplasm resources usually employs distinct and consecutive stages. This includes documentation of selected materials that should be performed in situ, development of asexual propagation protocols coupled with ex situ conservation of the produced material, and cultivation trials. The latter provides essential data regarding the potential for agronomical exploitation on a commercial scale as well as valuable materials for new crops and pre-breeding efforts [6,13,14]. Propagation-wise, successful asexual propagation protocols via cuttings with the use of external hormone application, such as indole-3-butyric acid (IBA) have already been demonstrated for several native phytogenetic resources in Greece and elsewhere [13,15,16,17,18,19]. Cultivation-wise, new native species originating directly from the wild flora of Greece or other Mediterranean areas have been recently assessed in pilot trials examining fertilization needs and testing different regimes [13,14,15,20,21,22,23]. Cultivation-wise, orchard management practices have long been studied in a plethora of mainstream tree crops across the Mediterranean and beyond, evidencing the benefits of diversified management systems compared to conventional ones, such as the increase of soil organic C and soil N through organic fertilization [24,25].

Species-wise, this study is focused on *Amelanchier ovalis* Medik (Rosaceae), which is commonly known as snowy Mespilus, serviceberry, or European juneberry (https://wfoplantlist.org/plant-list/taxon/wfo-0001003268-2022-12 accessed on 18 February 2023). This species naturally occurs throughout central and southern Europe, in sub-Mediterranean areas, and in south-west Asia [26]. *A. ovalis* is a Greek native deciduous shrub or small tree naturally occurring in bushland areas and along forest edges in rocky (limestone) terrain [27]. Natural tree heights can range from 1.5 to 3 m, and primary soft wood is covered with hair that turns brown as it develops. The leaves can be 2 to 7 cm long; they are ovate and serrate, with trichomes on the abaxial side when young [28]. *A. ovalis* in Greece is in flower from April through June depending on region, aspect, and altitude, showing impressive inflorescences of small hermaphrodite flowers with white petals. The flowers develop into small (5–15 mm in diameter) pomes of initially crimson/purple color that turn reddish-blackish during full maturity. The fruit taste is relatively mild due to its low acid and sugar content. However, its fruits are pleasantly edible and are associated with high nutritional and antioxidant value stemming from high levels of phenolic acids, anthocyanins, and flavonols [29,30,31]. *A. ovalis* is rather neglected and underutilized compared to the closely related North American *A. alnifolia* (Nutt.) Nutt. ex M. Roem., commonly known as Saskatoon berry. The latter formed an integral part of the traditional native American diet and medicine, with various parts of the plant being used to treat gastrointestinal ailments, infections, and complications of pregnancy and labor [32]. Recent studies, however, have revealed the beneficial effect of *A. ovalis* fruits on hematopoiesis as well as the antibacterial activity of the extracts from its leaves and branches [33,34,35]. Three subspecies of *A. ovalis* have been recorded in Greece (https://portal.cybertaxonomy.org/flora-greece/intro accessed on 18 February 2023), namely *A. ovalis* subsp. *cretica* (Willd.) Maire & Petitm. with East-Mediterranean range; *A. ovalis* subsp. *οvalis* with European range which concerns the current study; and *A. ovalis* subsp. *integrifolia* (Boiss. & Hohen) with almost circum-Adriatic range (http://ww2.bgbm.org/EuroPlusMed/PTaxonDetail.asp?NameCache=Amelanchier%20ovalis&PTRefFk=7300000 accessed on 18 February 2023). However, none is cultivated or agronomically exploited in any way. Additionally, any literature review regarding the asexual propagation of different *Amelanchier* spp. may only furnish information about the North American species *A. alnifolia* and *A. laevis* Wiegand. Both of these propagated with commercially acceptable results (>50% rooting) via the use of IBA on softwood cuttings under mist [36,37] or *A. canadensis* (L.) Medik., which showed better propagation performance [38]. To the best of our knowledge, there is no species-specific knowledge on the propagation and cultivation of *A. ovalis*. Only limited data on *A. ovalis* can be found, originating from in situ studies examining the climatic effects on the phenology during the growth cycle of wild European populations of *A. ovalis* in SE Spain [39,40]. 

In the above-mentioned framework, the current investigation aims to provide data for the first time on Greek *A. ovalis* germplasm for its sustainable agronomic exploitation. In particular, this study aimed at: (i) developing an asexual propagation protocol to secure the transferability of agronomical traits coupled with fast, reliable, and low-cost production of high volumes of plant material; (ii) launching a long-term pilot orchard-type cultivation trial with distinct fertilization management regimes tested under a diversified tree crop management framework (still ongoing); and (iii) evaluating the phytochemical potential of different organs of *A. ovalis* from cultivated material at the premises of the Balkan Botanic Garden of Kroussia (BBGK), Institute of Plant Breeding and Genetic Resources (IPBGR), Hellenic Agricultural Organization Demeter (ELGO-Dimitra) in northern Greece.

## 2. Results

### 2.1. Asexual Propagation of the Greek Native Germplasm

Hardwood-type propagation material collected from wild-growing populations of *A. ovalis* subsp. *ovalis* failed to root in all cases. Soft-wood cuttings produced limited rooting, which in most cases, was very low (≤5%) except for genotype GR-1-BBGK-04,2547, which produced comparatively higher rooting rates (20%) during preliminary trials with the application of 4000 ppm IBA in early summer (with no pre-treatment). As such, *A. ovalis* subsp. *ovalis* genotype GR-1-BBGK-04,2547 was selected for further experimentation.

Concerning the two experiments conducted in 2020, the cuttings from *A. ovalis* subsp. *ovalis* genotype GR-1-BBGK-04,2547 that was pre-treated with OFS failed to root in all cases in both experiments. The same holds true for the 0.25% powder ΙΒA treatment. In both experiments, rooting was achieved only with external hormone application without pre-treatment. In the first experiment, hormone treatments significantly affected rooting rates and the number of emerged roots on rooted cuttings (Table 1, *p* < 0.05). Rooting rates reached 83.3% after 28 days under 4000 ppm IBA and 2500 ppm NAA treatments without any significant differences in root number and length of rooted cuttings (Table 1, *p* < 0.05). Similarly, 2000 ppm IBA and 5000 ppm NAA showed 66.6% rooting with the latter showing a higher number of roots on rooted cuttings that surpassed all other treatments without, however, differences in root length (Table 1, *p* < 0.05). The highest IBA treatment (6000 ppm) showed the lowest rooting frequency (50%) (Table 1, Supplementary Materials Figure S1). In the second experiment, in which cuttings with higher levels of lignification were used (Supplementary Materials Figure S1), fewer cuttings managed to root. This was accompanied by the highest rooting treatments being 4000 ppm IBA and 2500 ppm NAA, both reaching 33.3% rooting after 30 days without differences in root length of rooted cuttings, followed by 2000 ppm IBA with 16.6% rooting. This occurred while the rest of the treatments (including the control) failed to root (Table 1, *p* < 0.05).

### 2.2. Total Phenolic Content and Antioxidant Activity

Regarding the Total Phenolic Content (TPC), no statistically significant difference was observed between the examined samples of leaves, twigs, and young fruits. Flowers, on the other hand, had a significantly lower TPC value (Table 2, *p* < 0.05). As far as the extracts’ antioxidant activity is concerned, no significant disparity between plant parts was observed, as all extracts exhibited remarkable radical scavenging capacity (Table 2). 

**Figure 1:**
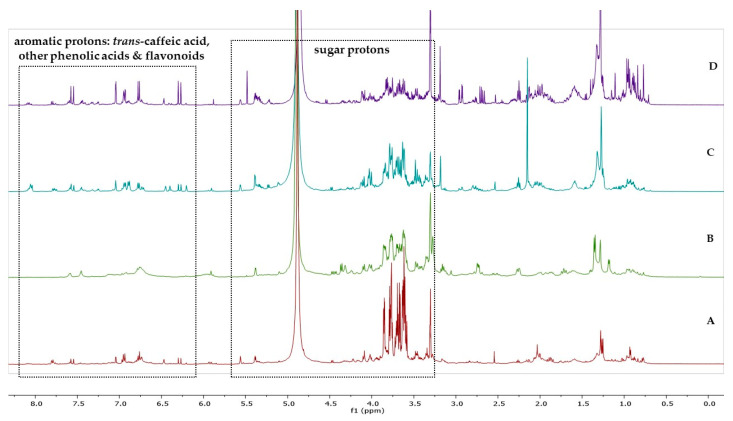
^1^H-NMR spectra of hydromethanolic extracts of (**A**) leaves, (**B**) twigs, (**C**) flowers, and (**D**) young fruits of *A. ovalis* subsp. *ovalis* genotype GR-1-BBGK-04,2547 (CD_3_OD, 500 MHz). Signals between 6.20–8.10 ppm correspond to aromatic protons of caffeic acid, other phenolic acids, and flavonoids, whereas signals between 3.20–5.55 ppm are attributed to sugar protons.

The ^1^H-NMR spectra of the extracts (Figure 1) revealed the presence of phenolic compounds (both as aglycones and glycosides) in all examined plant organs of *A. ovalis* subsp. *ovalis*. In specific, *trans*-caffeic acid derivates were identified by the characteristic pair of *trans*- olefinic proton signals at 7.56 and 6.29 ppm (d, *J* = 15.9 Hz), whereas signals that correspond to ABX systems and other aromatic protons (at 6.20–8.10 ppm), as well as signals between 3.20–5.55 ppm that correspond to sugar protons, attested to the presence of other phenolic acids and flavonoids. 

### 2.3. Field Cultivation Trial under Different Fertilization Regimes

**Figure 2:**
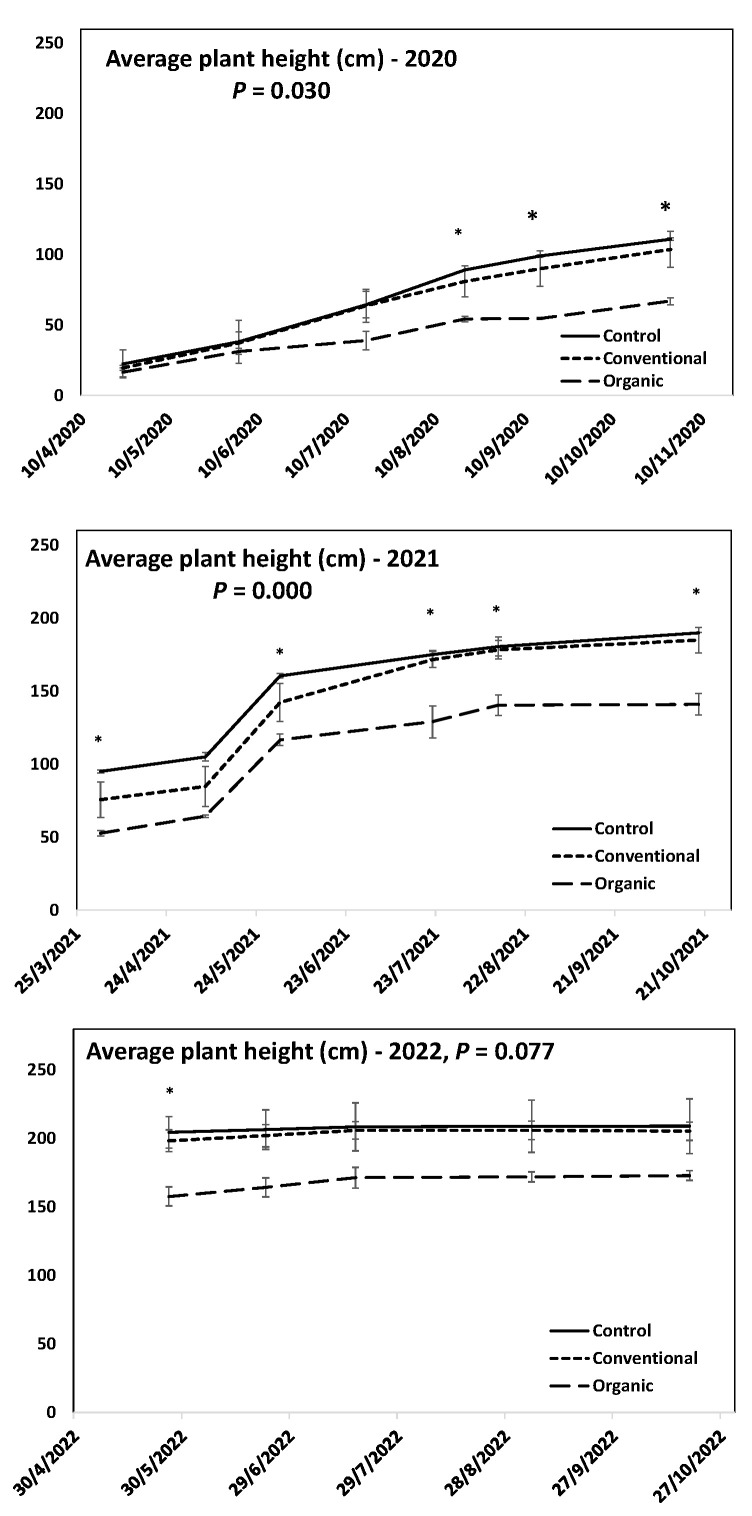
Plant growth patterns expressed as average plant height (cm) for *Amelanchier ovalis* subsp. *ovalis* genotype GR-1-BBGK-04,2547 during the three years of the pilot field trial (2020, 2021, and 2022) for the three fertilization treatments applied (control, conventional, and organic). Standard errors of the means are shown on the graphs (*p* < 0.05) as well as the respective *p* values of a repeated-measures ANOVA conducted on treatment effects over time (within-subjects effects) separately for each year (*p* < 0.05). Asterisks denote dates when significant differences between treatments were observed following discreet analyses for each measurement date via Tukey’s HSD mean comparison for each year separately (*p* < 0.05).

**Figure 3:**
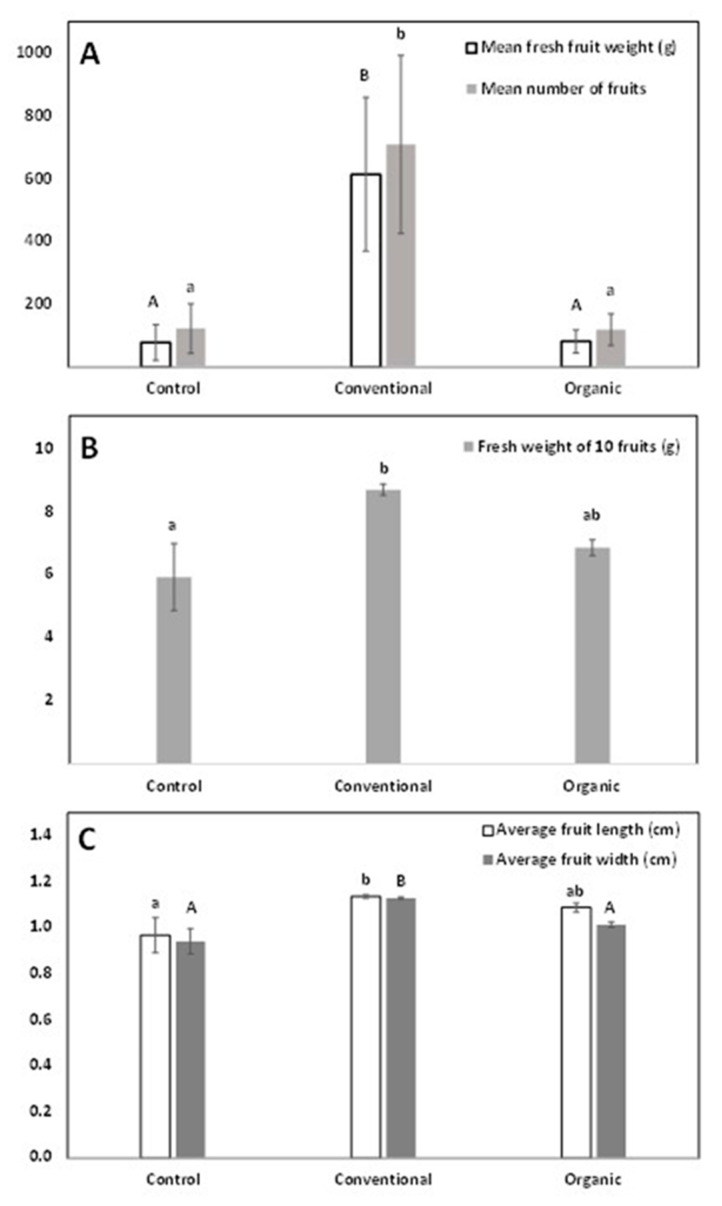
Fruit yield and morphometric data for *Amelanchier ovalis* subsp. *ovalis* genotype GR-1-BBGK-04,2547 during the 2022 pilot field trial for the three fertilization treatments applied (control, conventional, and organic). (**A**) Mean fresh fruit weight and mean number of fruits per treatment. Bars that do not share the same label letter are significantly different (Tukey’s HSD, *p* < 0.05); capital letters for fruit number, and lowercase letters for fruit fresh weight. (**B**) Mean fresh weight of ten randomly sampled fruits for each fertilization treatment. Bars that do not share the same label letter are significantly different (Tukey’s HSD, *p* < 0.05). (**C**) Average fruit length (cm) and average fruit width (cm) for each fertilization treatment. Bars that do not share the same label letter are significantly different (Tukey’s HSD, *p* < 0.05); capital letters for fruit width, and lowercase letters for fruit length. Standard errors of the means are shown on all graphs (*p* < 0.05).

Individuals of *A. ovalis* subsp. *ovalis* genotype GR-1-BBGK-04,2547 that were produced via cuttings were successfully established at the pilot field in IPBGR. Concomitantly with planting, the fertilization trial commenced. Overall, the results showed that the fertilization treatments had a significant effect on plant growth in terms of plant height. These effects seemed to change in magnitude at different times during each growing season and from one year to the next during the first three years of the trial, which is actually a big part of the young growth stage of the cultivated trees (Figure 2 and Figure 3).

During the first year of the trial (2020), the results showed that for the first five months after planting (March 2020), the individuals grew significantly in size, but the fertilization treatments used had no effect. This was statistically expressed as a significant effect of the time of the season (i.e., growth through time) but with no treatment effect, according to repeated measures ANOVA on plant height data that were recorded at regular intervals after planting (Figure 2, *p* < 0.05). However, after the 5th month (August 2020), significant differences between the organic fertilization treatment and the other two (control and conventional fertilization) were detected following discreet statistical testing for each measurement date, with organic fertilization exhibiting lower growth rates (Figure 2, *p* < 0.05). In August 2020, the trees were already 12 months old, and until the end of the season and the start of leaf shedding at the onset of dormancy, the trees reached 1.11 m, 1.04 m, and 0.67 m mean height in the control, conventional fertilization, and organic fertilization treatments, respectively (Figure 2, *p* < 0.05). 

Similarly, during the second year (2021), the time of season showed (as expected) a significant effect on plant height. All fertilization treatments showed similar growth patterns, with a significant increase in height between May and June/July 2021. In addition, organic fertilization was significantly lower throughout the growing season from March–October 2021 (Figure 2, *p* < 0.05). At the end of the 2021 growing season, the trees in the treatments reached 1.9 m, 1.85 m, and 1.41 m mean height in the control, conventional fertilization, and organic fertilization groups, respectively (Figure 2, *p* < 0.05).

**Figure 4:**
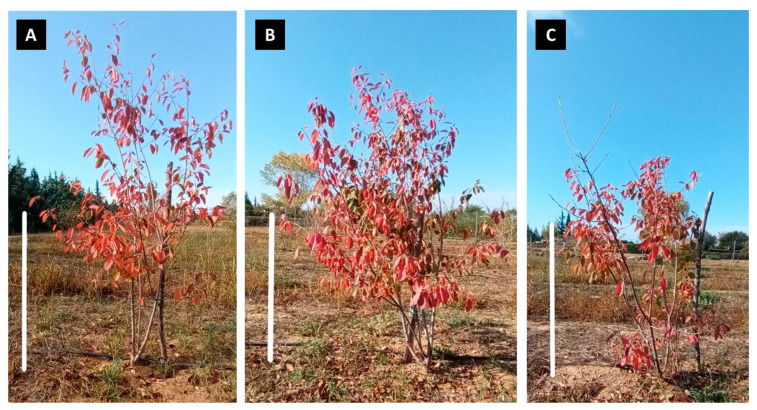
Representative growth of *Amelanchier ovalis* subsp. *ovalis* GR-1-BBGK-04,2547 trees in the pilot field (Thermi, Thessaloniki, Greece) during autumn 2022 with discolored leaves before shedding after different fertilization regimes applied ((**A**) control; (**B**) conventional fertilization; (**C**) organic fertilization). The bars in the photos represent 1 m.

During the third year (2022), it was found that the organic fertilization led to significantly lower growth at the beginning of the season in May 2022, whereas as the season progressed, no significant differences between treatments were detected. This was evidenced by similar growth patterns coupled with no time-of-season effects (Figure 2, *p* < 0.05). At the end of the 2022 growing season, the trees in each treatment reached 2.09 m, 2.05 m, and 1.73 m mean height in control, conventional fertilization, and organic fertilization, respectively (Figure 2 and Figure 4).

During the 2022 growth period, the trees produced fruits that were picked for the first time. According to the recorded fruit yield data, conventionally fertilized trees clearly produced more fruits, with 615 g of average fruit weight per tree compared to 83.4 and 78 g of average fruit weight per tree in organic fertilization and control, respectively (Figure 3, *p* < 0.05). Additionally, conventionally fertilized fruits were both heavier and larger in size than the control, based on a weight comparison of ten-fruit samples coupled with morphometric measurements of fruit length and width (Figure 3, *p* < 0.05). Moreover, 100 g of fresh fruits were randomly sampled from each treatment and were dried in an indoor dryer, resulting in 36.35 g of dry fruit per 100 g of fresh fruit for the control, 35.18 g of dry fruit per 100 g of fresh fruit for organic fertilization, and 25.05 g of dry fruit per 100 g of fresh fruit for conventional fertilization. The above measurement suggests that fruits produced from conventionally fertilized trees seem to contain more water.

## 3. Discussion

### 3.1. Asexual Propagation of Greek Native Amelanchier ovalis

The current propagation results of Greek *A. ovalis* subsp. *ovalis* germplasm indicates that soft-wood material with the least amount of lignification in combination with the external application of rooting hormone is probably the best method for cutting rooting induction. Similarly, evidence from the literature, albeit limited to scarce and old studies, has shown the positive effects of the application of hormone for rooting of soft-wood cuttings on germplasm of different *Amelanchier* spp. of both cultivated and wild origin [36,37,38]. Furthermore, the necessity of hormone application on soft-wood cuttings for rooting induction has recently been demonstrated on other Greek native wild germplasm of other species [8,14], including members of the Rosaceae family [13]. However, excessive levels of hormone application seem to reduce rooting capacity, which was more severe here in lignified cuttings (Table 1, Supplementary Materials Figure S1). Superfluous levels of hormone application on cuttings of woody species have known effects on rooting [41,42]. In a similar study on native Greek germplasm of *Sambucus nigra* accessions, superfluous levels of hormone application on cuttings have shown highly adverse effects [14]. A further factor that can affect adventitious root formation on cuttings can be the anatomical changes that take place during the growth and maturation of the stem tissue from which cuttings are excised since these changes are linked with the spontaneous emergence of roots from the meristematic cambium cell zones or cambium-derived callus areas outward and through the epidermis of the stem tissue of woody species [43,44,45,46]. During the current study, *A. ovalis* cuttings of young, soft tissue outperformed in rooting capacity cuttings of slightly older tissue with a higher level of lignification at all respective hormone application levels between the first and second experiments. Consequently, the current results support the superiority of soft-wood material with a minimum amount of lignification compared to hard-wood material during the asexual propagation of Greek *A. ovalis*. Such a prevalence of soft-wood material in cutting propagation has also been demonstrated elsewhere in the wild germplasm of members belonging to genera such as *Cornus* L., *Rosa* L., and *Prunus* L., among others [8,13,47,48].

The propagation results presented here are based on experimentation on a single genotype of Greek *A. ovalis* subsp. *ovalis* (GR-1-BBGK-04,2547), because the majority of the documented genotypes had very low to non-existent rooting potential. This suggests that the rooting of Greek wild-growing *A. ovalis* subsp. *ovalis*, apart from the cuttings’ lignification level and hormone application discussed above, may also depend on the genotype. In comparison to the current study, the effects of genotype on the rooting potential of cuttings have been observed in other members of the Rosaceae family, such as a broad spectrum of wild *Prunus* germplasm, where cuttings from different wild genotypes presented dramatic differences in rooting capacity, ranging in some cases from 0 to >50% rooting of different genotypes under matching treatments within each tested *Prunus* species as well as between different wild species of the genus *Prunus* [48]. Additional studies reported significant effects of genotype on the rooting of cuttings of *Prunus* rootstock germplasm tested both under mist and hydroponically [49,50,51]. Further studies on Rosaceae small trees have reported noteworthy differences in rooting of cuttings between wild genotypes of *Rosa canina* L. but also between wild genotypes of damask roses (*R.* x *damascena* Herrm.) [13,52]. A potential strategy that has been employed in other species to overcome the genotype problem in asexual propagation has been the method of grafting a desirable hard-to-root genotype onto a genotype with high rooting capacity for the steadfast production of stock material [53,54,55]. However, further research is suggested on the asexual propagation of the *A. ovalis* germplasm studied herein, including an assessment of grafting methods based on more extensive propagation data. 

Based on our results, it can be stated that the response of Greek *A. ovalis* cuttings to rooting is probably caused by several factors, such as the type and concentration of external hormone application, cutting type, season, and genotype. Other studies [56] have reviewed the control of adventitious rooting in cuttings as affected by internal (molecular) and external factors in a plethora of species. Such studies have demonstrated a multifactorial control of rooting via genetic, physiological, nutritional, physical, metabolic, and hormonal effects [56]. As a result, rooting of cuttings is a system-oriented concept in which genotype is involved at almost every stage [56]. 

### 3.2. Total Phenolic Content and Antioxidant Activity of Greek Native Amelanchier Ovalis

To the best of our knowledge, there is limited data concerning the phenolic profile and antioxidant activity of leaves, twigs, flowers, and young fruits of *A. ovalis* subsp. *Ovalis*, but also of other species of the genus for that matter, besides *A. alnifolia,* and all research lines tend to focus on mature fruits of members of the genus *Amelanchier*. The results concerning the TPC and radical scavenging activity as well as significant nutraceutical traits of leaves, twigs, flowers, and young fruits of Greek *A. ovalis* subsp. *ovalis* are therefore reported herein for the first time. In the study of Ekin et al. [57] hydroethanolic extracts from two *A. ovalis* subsp. *ovalis* leaf extracts from Turkey were screened (among other Rosaceae species) for their Acetylcholinesterase (AchE) and Butytylcholinesterase (BchE) inhibitory activity, alongside their in vitro antioxidant activity using FRAP assay, metal-chelation capacity by Fe^+2^-ferrozine test system and Radical scavenging effect against DMPD (N,N-dimethyl-p-phenylenediamine). Even though the extracts showed moderate to low enzyme-inhibitory activity (24.38 ± 3.26% and 0% for AchE, 19.99 ± 1.60% and 19.58 ± 1.67% for BchE), FRAP value (absorbance: 0.694 ± 0.02 and 0.793 ± 0.08,) and DMPD-antiradical activity (2.37 ± 1.72% and 8.40 ± 1.63%), their metal-chelating capacity was comparatively among the highest ones (14.51 ± 1.92% and 27.12 ± 0.46%). Nevertheless, the phytochemical profile of these extracts was not investigated, as only the most active samples were chosen for further analyses.

In another study, Lavola et al. [58], have analyzed the leaves, stems, and berries of four *A. alnifolia* (Saskatoon berry) cultivars growing in Finland using HPLC-DAD and HPLC-ESI/MS. The results showed that the leaves had a much higher polyphenol content compared to berries, both in terms of total phenolic acids (22.78 mg g^−1^ DW and 1.084 mg g^−1^ DW, respectively) and total flavonols (26.62 mg g^−1^ DW and 0.440 mg g^−1^ DW, respectively), thus indicating that leaves are expected to have a higher antioxidant activity than berries. In the same study, the stem extracts had a considerably different phenolic profile, as flavan-3-ol- and flavanone-derivatives were the main phenolic compounds (38% and 55% of total phenols, respectively). The aforementioned results are in concurrence with those of Tian et al. [59], indicating that *A. alnifolia* leaves may surpass branches and fruits in terms of total phenolic content (227.1 ± 0.7, 116.1 ± 2.5 and 49.8 ± 1.4 mg GAE 100 mL ^−1^, respectively, evaluated with the Folin-Ciocalteau assay), which, in turn, was only moderate when compared to leaf extracts from other berry plants. The extracts of the individual plant organs are also reported to exhibit strong radical scavenging activity, with the leaf extract being the most potent among them (88.8 ± 0.8%, 56.2 ± 1.1%, and 52.1 ± 4.6%, as measured at 10 min, using the DPPH assay). Conversely, Saskatoon berries are expected to be particularly rich in total phenolics. Similarly, Grygorieva et al. [60] have reported that *A. alnifolia* leaf extract has a moderate content of total polyphenols, total phenolic acids, and total flavonoids in comparison to other leaf extracts, though it may nonetheless exhibit potent antiradical activity. In the study of Męczarska et al. [61], on the other hand, Saskatoon berry extract was found to possess a higher TPC value than that of the leaf extract (406 ± 1.0 mg GAE g^−1^ DW versus 253 ± 4 mg GAE g^−1^ DW, as determined by the Folin-Ciocalteau method).

Zengin et al. [62] examined the phenolic profile (utilizing HPLC–MS/MS) and evaluated in vitro the total phenolic and total flavonoid content, as well as the antioxidant activity (utilizing phosphomolybdenum test, ABTS, DPPH, FRAP, CUPRAC, and ferrous chelating assays) of methanolic, aqueous, and ethyl acetate extracts of *A. parviflora* Boiss. subsp. *dentata* (Browicz) K.I. Chr. collected during the flowering season. In vitro results have shown that the methanolic extract has the highest total phenolic and total flavonoid content (125.28 ± 4.54 mg GAE g^−1^ and 49.14 ± 0.58 mg RE g^−1^, respectively) and the strongest antioxidant activity in all assays, except for the metal chelating assay, in which the ethyl acetate extract shows a greater potency.

Very high antioxidant potency was observed in the Greek *A. ovalis* subsp. *ovalis* genotype evaluated herein, with equally high antioxidant activity (AA) among the examined plant organs. However, in terms of total phenolic content (TPC), the extracts of leaves and twigs exhibited lower TPC values than their *A. alnifolia* counterparts, which can be attributed mostly to differences in the phytochemical profiles of the two species and environmental conditions as well between their regions of origin (North America, Mediterranean Greece). Furthermore, the flowers herein showed significantly lower phenolic content compared to young fruits, leaves, and twigs, albeit with equally high antioxidant activity. The phytochemical composition of the tested extracts, particularly the specific nature of the contained (poly)phenols, may result in differences between AA and TPC within the same plant tissue. Phenolic compounds are known to be potent antioxidants due to the presence of one or more phenol rings, alongside other functional groups, such as hydroxyl (-OH) or carboxyl (-COOH) groups, which enable polyphenols to act as radical scavengers, metal cation chelators, and hydrogen donors. Depending on their specific chemical structures, the antioxidant activity of the different classes of polyphenols can vary [31,59,63]. Differences in the chemical structure of phenols also affect the results of the Folin-Ciocalteu assay, as reported previously [64]. The results of the NMR spectrometry corresponded to the observed composition of the extracts in terms of antioxidant potency. Existing literature concerning the phytochemical composition of *A. ovalis* corroborates the current results, reporting the presence of phenolic acids (both hydroxybenzoic and hydroxycinnamic derivatives), depsides (such as chlorogenic acid), flavonol glycosides (mainly quercetin-type), and anthocyanins (mainly cyanidin-type) [29,30,31,32,65]. In a previous study [30], mature *A. ovalis* fruits were found to contain the highest content of total polyphenols (356.20 mg 100 g^−1^) among the examined species (*A. alnifolia, A. ovalis,* and *A. canadensis*), especially in terms of total anthocyanin (203.23 mg 100 g^−1^) and total flavonol (33.26 mg 100 g^−1^) content. Moreover, some studies [31] report the strong radical scavenging activity of the Siberian *A. ovalis* berry extract as evaluated in vitro employing the ABTS assay, as well as its ex vivo activity against H_2_O_2_-induced oxidative stress in *Saccharomyces cerevisiae* Y-564 yeast. The current results on the antioxidant profile of Greek *A. ovalis* in different plant organs may suggest that the synthesis and transport of secondary metabolites with antioxidant activity may be systemic (of a whole-plant nature), at least for young, developing tissues. To this end, Green and Mazza [66] have found that the ripening of *A. alnifolia* fruits may lead to an increase in total anthocyanins and total phenolics. On the other hand, some studies [67] report a decrease in total phenolics and an increase in total anthocyanins, especially through the later stages of maturation. Undoubtedly, the need for further investigation of secondary metabolites and their health-promoting activities as a rich source of nutraceuticals is strongly suggested.

### 3.3. Pilot Orchard-Type Cultivation of Greek Native Amelanchier ovalis

The acclimatized new plants of Greek *A. ovalis* subsp. *ovalis* (GR-1-BBGK-04,2547 produced via the asexual propagation attempts discussed above) were successfully established in a pilot field cultivation trial. The 2020-initiated (still ongoing) orchard-type trial has provided up-to-date data on the establishment of *A. ovalis* subsp. *ovalis* individuals during the first three years of their juvenile growth under distinct fertilization treatments, including organic fertilization, applied gradually throughout every growing season aimed at its novel assessment as a diversified tree crop as opposed to traditional and conventionally managed tree crops. Considering the observed phenological progression of Greek *A. ovalis* subsp. *ovalis* trees over each studied growing season, their vegetative growth in the pilot field trial was active almost throughout each season from April through October for all experimental treatments applied. In contrast, the vegetative growth studied in Spanish wild-growing populations of *A. ovalis* was active mainly from April to May/June, thus presenting limited phenological development and nutrient retention in leaves over the dry summer period [39]. This higher growth capacity observed in the pivotal orchard-type trial may be linked to the fact that trees were irrigated throughout their growing season, contrasted to wild-growing trees with natural water seasonality and scarcity in the Mediterranean context [68]. However, flower bud formation, flowering, and fruit set in the current field trial took place during the same period as its wild *A. ovalis* counterparts studied in Spain under similar climatic conditions (Mediterranean), albeit at a higher altitude in the case of Spain [39]. This observation may stem from the genetic control of reproductive transition as opposed to vegetative growth, with the latter probably being more affected by environmental conditions such as water availability. Higher levels of reproductive phenological synchronization than vegetative phenophase synchrony among wild-growing populations, both inter- and intra-species, including *A. ovalis*, have been observed in Mediterranean environments with continental features, attributing vegetative asynchrony to resource availability differences among habitats [40].

According to the current data, both control and conventional fertilization demonstrated higher growth rates expressed as greater plant height, albeit under similar patterns compared to organic fertilization during the first two years. In the third year, however, the vegetative growth rates were similar between fertilization treatments. This observation may be linked to the fact that during the third year, the plants entered the reproductive stage of their life cycle and produced fruits, thus re-distributing their assimilate transport toward reproductive growth and ameliorating differences in vegetative growth [69]. The produced fruit load in the third year was different between fertilization treatments with a conventional fertilization regime, which was rich in N throughout, delivering higher fruit production without significantly higher vegetative growth rates. Fertilization studies on cultivated germplasm of mainstream Rosaceae tree crops such as pears (*Pyrus* spp.) and almonds (*Prunus amygdalus* Batch) have demonstrated that conventional fertilization, and more specifically N fertilization, when applied up to specific thresholds, may enhance soil N concentration and fruit yield, affecting fruit quality [70,71]. On the other hand, organic fertilization via green manure in almonds has been suggested to be applied in combination with some form of tillage between rows under semi-arid conditions for adequate fruit production [72]. Organic fertilization in the form of farmyard manure has been studied in other typical Mediterranean tree crops like olives (*Olea europaea* L.), where it was shown to enhance soil fertility via an increase in organic C content while positively affecting production [73]. However, in the current study, organic fertilization was applied in the form of standardized organic fertilizers containing, among others, a plethora of elements, organic acids, amino acids, humic acid, and nitrogen, and was applied gradually in targeted applications over each growing season. To the best of our knowledge, this type of fertilization was applied here for the first time, and further observations over the next few years are suggested to draw safe conclusions.

Overall, the current data on Greek native *A. ovalis* subsp. *ovalis* as affected by fertilization regimes within a pivotal commercial cultivation setting have not shown differences between conventional fertilization and control in early tree establishment and growth as a potential new crop. In addition, young tree establishment and growth may also be dependent on soil fertility properties such as organic matter and N concentration [24]. On the other hand, external fertilization inputs did deliver higher fresh fruit production herein, with conventionally fertilized trees producing more fruits that were both heavier and larger in size than the other two treatments (control and organic fertilization). However, the fruits produced under conventional fertilization showed higher water content. In general, high fresh fruit water content in relation to fruit size may affect fruit quality in terms of soluble carbohydrates, free amino acids, and desirable secondary metabolites due to a dilution effect. The alteration of the biochemical profile of significant metabolites in relation to fruit water content and size has been repeatedly marked in Rosaceae soft fruits and tree crops as well as in grapes [74,75,76,77]. As such, in cases where high fresh fruit volumes of *A. ovalis* subsp. *ovalis* are desirable, conventional fertilization may deliver positive results. However, from the current data, it is unclear whether the higher fresh fruit production in terms of fruit size, weight, and water content takes a toll on the nutritional quality of the fruit in terms of desirable antioxidant/secondary metabolite concentrations. The latter suggests the need for further research on Greek *A. ovalis* fruits’ phytochemical profile under the pivotal commercial cultivation setting.

## 4. Materials and Methods

### 4.1. Documentation of Plant Material

**Figure 5:**
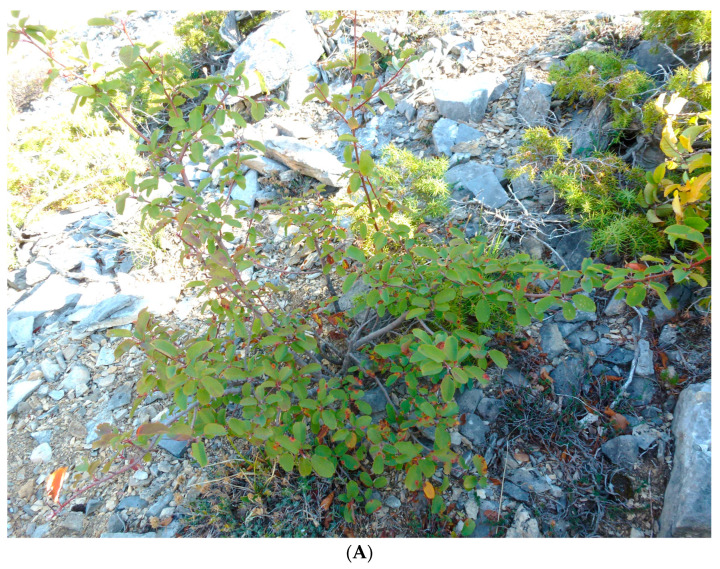

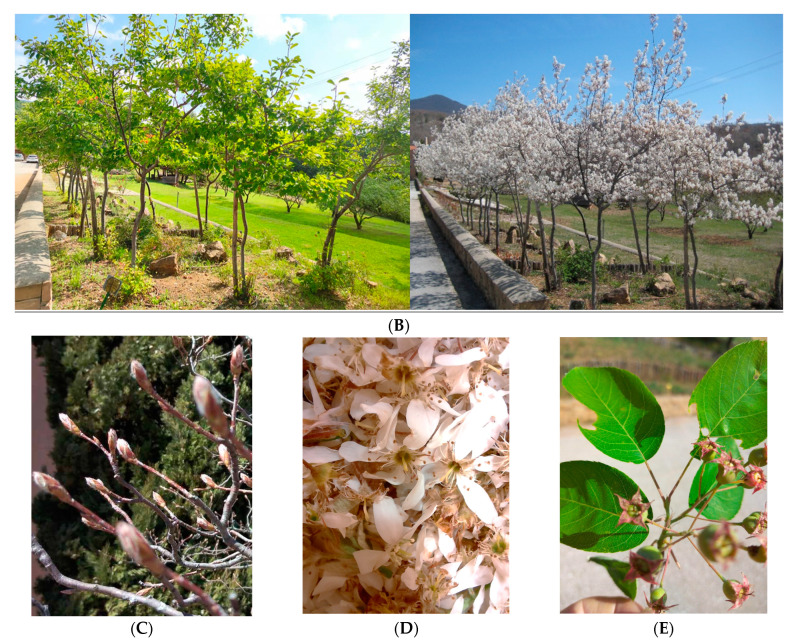
(**A**) Appearance of in situ adapted wild-growing young individual of *Amelanchier ovalis* subsp. *ovalis* GR-1-BBGK-19,928 from Mt. Tzena, Pella (northern Greece) thriving in an inclined rocky substrate; (**B**) Mature trees of *Amelanchier ovalis* subsp. *ovalis* GR-1-BBGK-04,2547 from Mt. Tzena (Pella, northern Greece) acclimatized ex situ at the grounds of the Balkan Botanic Garden of Kroussia (600 m above sea level); Young twigs (**C**), flowers (**D**), leaves and young fruits (**E**) of ex situ cultivated *Amelanchier ovalis* subsp. *ovalis* GR-1-BBGK-04,2547 from Mt. Tzena used for phytochemical evaluation.

**Figure 6:**
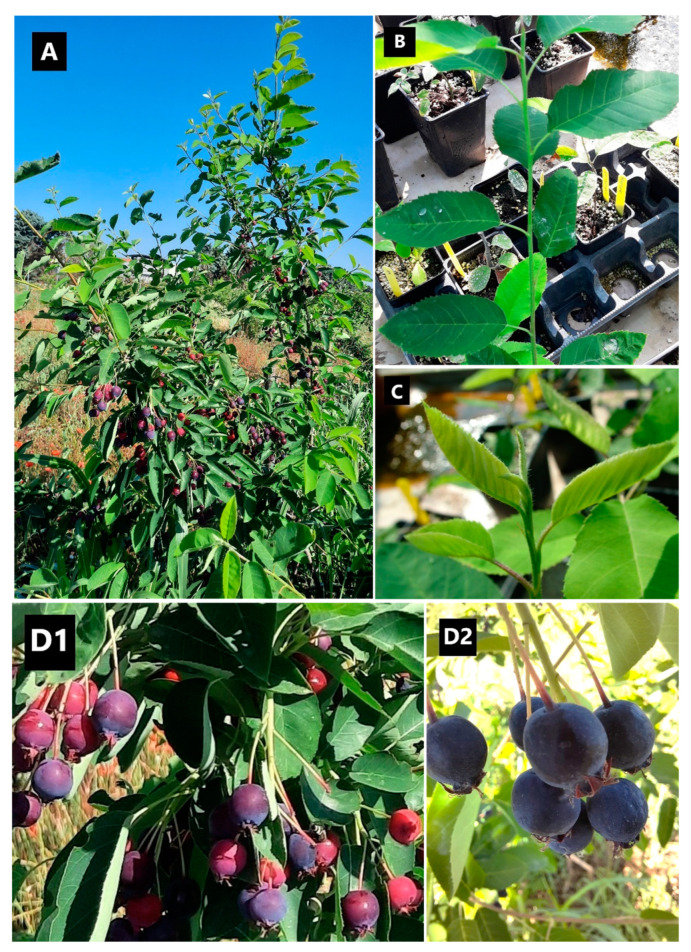
Morphology of ex situ acclimatized Greek native germplasm of *Amelanchier ovalis* subsp. *ovalis* GR-1-BBGK-04,2547. (**A**) Representative appearance of an ex situ grown adult plant, (**B**) Mature leaves, (**C**) Apical meristem with young leaves, (**D1**) Developing fruits (pomes), (**D2**) Typical coloration of mature fruits.

**Figure 7:**
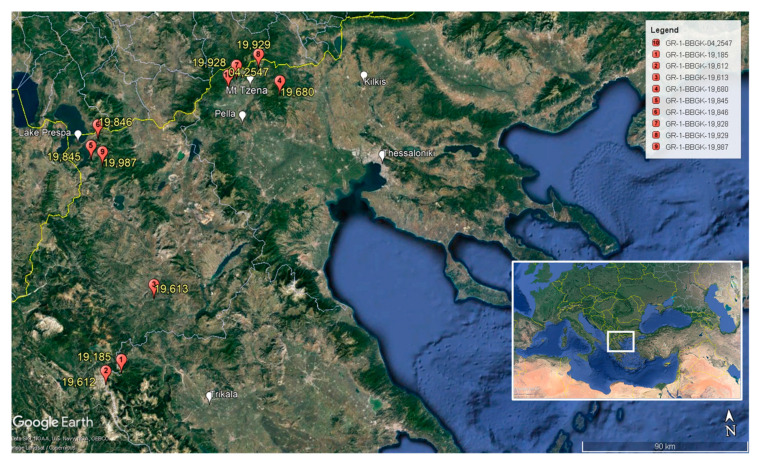
Geographical distribution of Greek native *Amelanchier ovalis* subsp. *ovalis* wild-growing populations sampled across northern Greece. The last 5 or 6 digits of the IPEN accession number are given for each population on the map, with full IPEN accession numbers shown in the legend. For localities, geographical coordinates, collection details, and types of collected material see Table 3.

Wild-growing populations of *A. ovalis* subsp. *ovalis* were initially located in Mt. Tzena (prefecture of Pella) in 2004 and after targeted botanical expeditions during 2018–2019 in different regions of northern Greece, including Mt. Tzena (Table 3, Figure 5A). The plant material was collected under a special permit to the IPBGR, ELGO-Dimitra (Permit 82336/879 of 18 May 2019, and 26895/1527 of 21 April 2021) issued by the Greek Ministry of Environment and Energy. The collected plant material consisted of primary soft-wood cuttings and hardwood cuttings of both apical and sub-apical stem parts for propagation, and the materials were taxonomically identified using diagnostic keys [78]. Each collected sample from geographically isolated populations represented a distinct genotype, which was assigned a unique IPEN (International Plant Exchange Network) accession number given by the BBGK. The investigation resulted in ten collected population samples from different habitats across northern Greece (Table 3). The collected materials were transferred to the laboratory of IPBGR for further handling and use in experimentation (Figure 5, Figure 6 and Figure 7).

### 4.2. Preliminary Propagation Trials of the Collected and Documented Greek Germplasm

In preliminary trials, the material from the collected population samples of *A. ovalis* subsp. *ovalis* was assessed for asexual propagation potential. Each population sample’s cuttings were prepared for rooting in propagation trays with peat (Klasmann, KTS 1): perlite at 1:3 *v*/*v* under automated misting within a greenhouse at ambient temperature and relative humidity (RH) maintained at >85%. External indole-3-butyric acid (IBA) hormone was applied in all cases via a quick dip (10 sec) of the basal part of the cuttings into the hormone solution. The hormone concentrations used varied from 2000 to 4000 ppm for soft material and up to 10,000 ppm for hardwood cuttings. The rooting of cuttings was assessed weekly. In cases where successful propagation was achieved, the produced plants were consecutively transplanted from trays to 1 L pots for one week and then to 3 L pots for plant establishment using a mixture of peat (Klasmann, KTS 2): perlite at 3:1 *v*/*v* and were watered regularly.

### 4.3. Propagation Experiments with Greek Germplasm

The population sample GR-1-BBGK-04,2547 was qualified for further experimentation as it was the genotype that performed better in preliminary trials. Two consecutive asexual propagation experiments were conducted in 2020. The second experiment mirrored the first in design and treatment structure but with a different starting material. The first experiment was set for May 2020, and the second was set for July 2020. For the first experiment, fresh, leafy, primary soft-wood cuttings (internode sections) with 2–3 buds and two fully developed leaves were taken from mother plants of the genotype GR-1-BBGK-04,2547. For the second experiment, fresh, leafy cuttings were taken from the primary wood of the same mother plants, but at a later developmental stage than the first experimental material, resulting in cuttings with a more advanced level of lignification compared to those used in the first experiment (see Supplementary Materials Figure S1). Including control, a plethora of thirteen external hormone treatments were applied in both experiments. Indole-3-butyric acid (IBA) was used at concentrations of 2000, 4000, and 6000 ppm (dissolved in 50% ethanol), and 1-Naphthaleneacetic acid (NAA) was also used at concentrations of 2500 and 5000 ppm (dissolved in 50% ethanol) via a quick dip (10 sec) of the basal part of the cuttings into the hormone solution. In addition, most of the above treatments were also applied in combination with a pre-treatment of a 30-min dip of the cuttings’ base into a commercially available organic fertilizer solution before the hormone dip. The organic solution used contained 2.5% water-soluble copper, 1.5% organic matter, and 3% water-soluble nitrogen, as used in commercial applications for seedlings and young plantlets for root enhancement and protection against root rots. In Table 4, the details of the applied propagation treatments are given. The cuttings were set for rooting in propagation trays with peat (Klasmann, KTS 1): perlite at 1:3 *v*/*v* under automated mist within a greenhouse at ambient temperature with relative humidity (RH) maintained >85%.

The cuttings were assessed for rooting weekly for four weeks. After this period, the rooted cuttings were taken out of the trays, and rooting frequency per treatment, root number, and average root length per cutting were recorded. Concurrently, rooted cuttings were transplanted in 1 L pots using a mixture of peat (Klasmann, KTS 2): perlite at 3:1 *v*/*v* and were kept for the first two weeks within a greenhouse with automated irrigation for plant establishment.

### 4.4. In Vitro Assessment of the Total Phenolic Content and Antioxidant Activity in Different Organs of the Greek Germplasm

#### 4.4.1. Extracts from Plant Material

Flowers, leaves, and twigs collected during spring and young fruits collected in early summer 2020 were firstly air-dried in the dark at room temperature and then comminuted and extracted separately with aqueous methanol as follows: 1 g of dried and comminuted plant tissue was extracted with 30 mL of MeOH_(aq)_ 70% *v*/*v* for 10 min in a sonicator bath at room temperature. The extracts were filtered through a paper filter, and the sediment was re-extracted a second time with an additional 30 mL of the extraction solution. The filtrates were combined and dried in vacuo (40 °C, Rotavapor^®^ R-210, Büchi, Labortechnik, Flawil. Switzerland). ^1^H-NMR (Nuclear Magnetic Resonance) spectra of all extracts were recorded in an AGILENT DD2 500 NMR spectrometer (20 mg of extract dissolved in 500 μL of CD_3_OD) operating at 500 MHz. Chemical shifts are reported in ppm (*δ*). Before the in vitro analyses, the extracts were reconstituted with DMSO at a concentration of 5 mg mL^−1^.

#### 4.4.2. Determination of Total Phenolic Content (TPC)

The total phenolic content of the individual plant organs was determined using the Folin-Ciocalteu method [79] as follows: 20 μL of the extract was mixed with 2.500 μL of deionized water and 400 μL of Folin-Ciocalteu reagent (F9252, Sigma-Aldrich, Darmstadt, Germany). After an initial incubation in the dark for 8 min at room temperature, 500 μL of Na_2_CO_3_ 7% (*w*/*v*) was added, and the reaction mixture was incubated for 30 min in the dark at 40 °C. The samples’ absorbance was measured at 750 nm using a UV-vis spectrophotometer (UV-1700 PharmaSpec, Shimadzu, Kyoto, Japan) and their TPC was calculated using a gallic acid standard curve (0–1.5 mg mL^−1^, R^2^ = 0.947). The results were expressed as mg of gallic acid equivalents per L of extract (mg GAE L^−1^). The assay was performed in triplicate, and the results represent the mean of three replications ± SD for each sample.

#### 4.4.3. Evaluation of Antioxidant Activity (AA)

The extracts’ antioxidant activity was evaluated using the DPPH (2,2-diphenyl-1-picrylhydrazyl) method, following the modified protocol of Risaliti et al. [80] as follows: an aliquot of DPPH (D211400, Sigma-Aldrich, Darmstadt, Germany) solution in MeOH (0.1 mM) was added to 20 μL of extract, and the mixture was left to stand in the dark at room temperature for 30 min. The absorbance of the reaction mixture was read at 517 nm using a UV-vis spectrophotometer (UV-1700 PharmaSpec, Shimadzu, Kyoto, Japan). The results were expressed as % Radical Scavenging Activity (% RSA), calculated using the following formula:

(1) $$\%\;RSA=[\left(A_{o}-A_{s}\right)/A_{o}]\times 100$$

where *A_o_* is the absorbance of the control, and as is the absorbance of each sample. The assay was performed in triplicate and the results represent the mean of three replications ± SD for each sample.

### 4.5. Pivotal Field Cultivation Trial of the Greek Germplasm

The pilot field trial of the *A. ovalis* GR-1-BBGK-04,2547 genotype was set on the grounds of the IPBGR, ELGO-Dimitra in Thermi near Thessaloniki, Greece (40.534934 N, 23.002401 E, 40 m elevation). The topsoil was of medium composition, loamy with 34% clay and 48% sand, and slightly alkaline with 1.37% organic matter. The main macronutrient concentrations for a 0–30 cm depth sample were 19 ppm N, 3 ppm P, 200 ppm K, 211 ppm Mg, and >2000 ppm Ca.

The fertilization treatments applied were: (a) no fertilization (control); (b) conventional crop fertilization and (c) organic crop fertilization. Specific application regimes were empirically calculated and designed for conventional and organic fertilization treatments based on the soil analysis, and these were applied throughout each growing season (see Supplementary Materials Table S1). Conventional fertilization was applied in the form of commercial inorganic granulated fertilizers containing N, P, Zn, S, Fe, and B. Organic fertilization was applied in the form of standardized organic fertilizers containing, among others, a plethora of elements, organic acids, amino acids, humic acid, and nitrogen. The details of the fertilization regimes and their application intervals are given in Supplementary Materials Table S1. During the growing season, all plants were drip-irrigated weekly with 1.92 L/h. The establishment of the trees was assessed through above-ground plant growth in terms of plant height, which was recorded at regular intervals from the beginning of the trial (planting) across each annual growing season (April–October) for the entirety of the three years of the study (2020–2022). Furthermore, when the trees began to bear fruit in the third year after planting, fruit production was measured in terms of fruit number, fruit weight, and fruit morphometric dimensions of length and width per tree. 

The climatic conditions of the region can be described as Mediterranean with continental features. The annual mean temperature during the three years of the study fluctuated between 16.9 and 17.8 °C with annual precipitation showing higher variation between 338 and 423 mm (Supplementary Materials Figure S2). The area typically is characterized by a warm season from May to September with average monthly temperatures >20 °C and maximum temperatures above 30 °C. At the same time, the months between May and September were also the driest months with the lowest amounts of precipitation for 2020 and 2021, whereas, in 2022, rainfall was more evenly distributed across the year (Supplementary Materials Figure S2). The trial was set under standard orchard practice techniques implemented in Greece. In March 2020, eight-month-old, acclimatized, cutting-originated plants were planted at the pilot field at N–S orientation.

### 4.6. Experimental Design and Statistical Analysis

Propagation-wise, both experiments on the *A. ovalis* GR-1-BBGK-04,2547 genotype followed a completely randomized design with 13 hormonal treatments and six replicate cuttings per treatment. The rooting attributes (root number and average root length per cutting) data for the first propagation experiment were subjected to analysis of variance (GLM-ANOVA) to establish treatment effects. The means were compared using Tukey’s HSD post hoc test at the a = 0.05 significance level. For the second propagation experiment, rooting attribute data were found not to be homogenous (Levene’s test), and as such, the non-parametric Kruskal-Wallis test was applied to assess treatment effects while means were compared using Dunnet’s T3 test (a = 0.05). In addition, the observed rooting frequencies for each treatment were compared in pairs for each experiment separately via consecutive Pearson Chi-Square tests (a = 0.05).

Cultivation-wise, a completely randomized design was followed, which included three distinct fertilization treatments (including a control) with five replicate plants per treatment. Treatment effects on plant height data measured over time were assessed through repeated measures ANOVA for each growing season separately (a = 0.05) to conclude whether the observed differences resulted over time, were purely due to treatment effects, or both. In addition, a GLM-ANOVA was conducted separately for each measurement date and each season, coupled with a mean comparison through Tukey’s HSD post hoc test (a = 0.05), to pinpoint any specific treatment differences at different stages of plant development.

The phytochemical analyses were measured in triplicate and a mean coupled with its standard deviation (±S.D.) was calculated in each case. Phytochemical data were analyzed using one-way ANOVA and means were compared using Tukey’s HSD post hoc test (a = 0.05).

All analyses were conducted using the IBM-SPSS 23.0 software (IBM Corp., Armonk, NY, USA), and graphs were drawn using Microsoft Excel.

## 5. Conclusions

The current investigation sets a groundwork framework for the sustainable agronomic exploitation of the Greek native germplasm of *Amelanchier ovalis* subsp. *ovalis* by documenting a selected genotype originating from Greek native wild-growing populations. This was carried out using a multifaceted approach that included: (i) facilitation of its sustainable utilization through the development of a species-specific asexual propagation protocol; (ii) agronomic evaluation in a pilot field cultivation trial; and (iii) comparative phytochemical evaluation of plant organs from the cultivated germplasm.

The above-mentioned steps are presented for the first time in this study. This provides substantial data for future applied research attempts on the sustainable exploitation of this noteworthy genetic resource with significant antioxidant capacity, which makes it a potential superfood candidate with additional ornamental and medicinal value.

## Figures and Tables

**Table 1:** Overview of the rooting results for *A. ovalis* subsp. *ovalis* genotype GR-1-BBGK-04,2547 with rooting frequencies and the corresponding rooting attributes in terms of root number and average root length (±SEM, *p* < 0.05) of the rooted cuttings for each hormone treatment in the two experiments conducted in 2020. Cuttings in both experiments were leafy soft-wood sections of primary stem growth with cuttings for the second experiment having a more advanced level of lignification. Rooting conditions were under automated mist within a greenhouse at ambient temperature and relative humidity (RH) was maintained at >85%. None of the combinations of hormone treatment and pre-treatment were successful. Thus, only the results from treatments under which cuttings managed to root in either of the two experiments are shown. The control treatments (i.e., no hormone treatment or pre-treatment application), failed to root in both experiments.

Experiment	Hormone Treatment	(%) Rooting	Mean Root Number *	Mean Root Length (mm)
1	Control	0.0	-	-
	2000 ppm IBA	66.6	6.25 (±0.75) a	31.27 (±5.88) A
	4000 ppm IBA	83.3 ^†^	6.40 (±2.03) a	22.26 (±4.23) A
	6000 ppm IBA	50.0	6.66 (±0.33) a	34.74 (±6.13) A
	2500 ppm NAA	83.3 ^†^	6.20 (±0.58) a	24.24 (±3.27) A
	5000 ppm NAA	66.6	36.50 (±16.65) b	18.42 (±3.69) A
2	Control	0.0	-	-
	2000 ppm IBA	16.6	6.00 (±0.00) **	33.50 (±0.00)
	4000 ppm IBA	33.3 ^†^	11.00 (±0.00)	31.18 (±4.54) A
	6000 ppm IBA	0.0	-	-
	2500 ppm NAA	33.3 ^†^	9.00 (±0.00)	18.11 (±2.66) A
	5000 ppm NAA	0.0	-	-

The † symbol denotes the highest rooting frequencies following pairwise comparisons of the observed rooting frequencies via Pearson X^2^ tests (a = 0.05) conducted for each experiment separately. * Values within each column for each experiment that do not share the same letter are significantly different (Tukey HSD, *p* < 0.05 for the first experiment, Dunnet’s T3 test *p* < 0.05 for the second experiment), with lowercase letters for root number and capital letters for average root length. The analysis was conducted for each experiment separately. ** In cases where only one replicate cutting managed to root, the standard error of the means for root number and length is 0.0 because they stem from a single value, as is the standard error of the means for root numbers that stem from two identical values; as such, those means are not included in the post-hoc test.

**Table 2:** Total phenolic content (TPC—mg GAE L^−1^ extract) and antioxidant activity (AA) expressed as Radical Scavenging Activity (% RSA) were measured in four different plant organs of *A. ovalis* subsp. *ovalis* native Greek genotype GR-1-BBGK-04,2547.

Plant Organ	Phytochemical Attribute	
	Total Phenolic Content (mg GAE L^−1^)	Antioxidant Activity (%RSA)
Leaves	37.639 (4.432) a	93.239 (1.609) a
Twigs	32.476 (3.966) a	93.431 (0.626) a
Flowers	9.468 (1.675) b	92.056 (1.596) a
Young fruits	33.214 (3.108) a	93.136 (1.541) a

Values represent mean values with standard deviation in parentheses (SD) of samples analyzed in triplicate (n = 3); values within the same column that do not share the same letter are significantly different (Tukey post-hoc test, *p* < 0.05).

**Table 3:** IPEN (International Plant Exchange Network) accession numbers assigned to the collected samples of Greek native *Amelanchier ovalis* wild-growing populations, with collection details and the types of material collected.

No	IPEN Accession Number	Greek Prefecture	Area	Coordinates (HGRS87/EGSA87) (Lat, Lon)	Altitude (m)	Collected Material *
1	GR-1-BBGK-19,185	Thessaly	Mt Lakmos, Trikala	39.667963, 21.135741	1984	HC
2	GR-1-BBGK-19,612	Thessaly	Mt Lakmos, Trikala	39.650822, 21.150658	1763	SC
3	GR-1-BBGK-19,613	Thessaly	Venetikos river, Trikala	40.052361, 21.481166	468	HC, SC
4	GR-1-BBGK-19,680	Macedonia	Mt Paiko, Kilkis	40.98824, 22.34423	750	HC, SC
5	GR-1-BBGK-19,845	Macedonia	Lake Prespa, Oxia	40.73076, 21.12622	1196	HC, SC
6	GR-1-BBGK-19,846	Macedonia	Lake Prespa, Oxia	40.72997, 21.11763	1249	HC, SC
7	GR-1-BBGK-19,928	Macedonia	Mt Tzena, Pella	41.12088, 22.21743	1152	HC, SC
8	GR-1-BBGK-19,929	Macedonia	Mt Tzena, Pella	41.12242, 22.21924	998	HC, SC
9	GR-1-BBGK-19,987	Macedonia	Lake Prespa, Oxia	40.72767, 21.11599	1282	HC, SC
10	GR-1-BBGK-04,2547	Macedonia	Tzena, Pella **	41.123857, 22.184903	1120	HC, SC

* SC: Soft-wood stem cuttings for propagation; HC: Hard-wood stem cuttings for propagation. ** Ex situ conserved in the Balkan Botanic Garden of Kroussia.

**Table 4:** Overview of the applied hormonal treatments involving indole-3-butyric acid (IBA), 1-Naphthaleneacetic acid (NAA), and pre-treatments with a commercial organic fertilizer solution (OFS) on the cuttings’ experiments of the Greek *Amelanchier ovalis* subsp. *ovalis* population sample GR-1-BBGK-04,2547. All hormone concentrations in the two trials except the 0.25% powder were dissolved in 50% ethanol. In combined treatments, the dip in OFS was for 30 min followed by a dip of the basal part of the cutting in the hormone solution for 5–7 sec.

No	Pre-Treatment	Treatment
1	-	Control
2	-	2000 ppm IBA
3	-	4000 ppm IBA
4	-	6000 ppm IBA
5	-	2500 ppm NAA
6	-	5000 ppm NAA
7	5% OFS *	2000 ppm IBA
8	5% OFS *	4000 ppm IBA
9	5% OFS *	2500 ppm NAA
10	5% OFS *	5000 ppm NAA
11	10% OFS *	2000 ppm IBA
12	10% OFS *	4000 ppm IBA
13	-	0.25% powder ΙΒA

* 2.5% water soluble copper + 1.5% organic matter + 3% water soluble nitrogen.

## Data Availability

All data supporting the results of this study are included in the manuscript and/or Supplementary Materials, and datasets are available upon request.

## Supplementary Materials

The following supporting information can be downloaded at: https://www.mdpi.com/article/10.3390/plants12051142/s1. Table S1: Overview of the conventional and organic fertilization regimes applied in the pilot field trial of *Amelanchier ovalis* subsp. *ovalis* GR-1-BBGK-04,2547 genotype during the experimental period (2020–2022). Figure S1: Indicative photos of cutting propagation of the Greek native genotype of *Amelanchier ovalis* subsp. *ovalis* GR-1-BBGK-04,2547. (A1) Prepared soft-wood cuttings (experiment 1); (A2) Prepared cuttings for the second experiment; (B) Rooting results (experiment 1) across eight of the thirteen applied treatments, i.e., control, 2000 ppm indole-3-butyric acid (IBA), 4000 ppm IBA, 6000 ppm IBA, 2500 ppm 1-Naphthaleneacetic acid (NAA), 5000 ppm NAA, and the failed treatments 0.25% powder IBA and 2000 ppm IBA pre-treated with 5% organic fertilizer solution (OFS); (C1): Rooted individuals; (C2): Acclimatized, ex situ raised plants. Bars in photos represent 10 cm; Figure S2: Temperature and rainfall variation during the experimental period (2020, 2021, 2022) with average temperature and the total precipitation for each year) of the pilot field trial study in the experimental grounds of Thermi, Thessaloniki, Greece (elevation 40 m), presented yearly as monthly average temperature (°C, T) patterns coupled with mean monthly high and mean monthly low temperatures (°C, T) and accumulated total monthly precipitation (ppt, in mm) patterns.
