# Supplementary material for: From the Wild to the Field: Documentation, Propagation, Pilot Cultivation, Fertilization, and Phytochemical Evaluation of the Neglected and Underutilized Amelanchier ovalis Medik. (Rosaceae)
Source: Plants (Basel). 2023 Mar 2;12(5):1142. doi: 10.3390/plants12051142 (PMC10006941; doi:10.3390/plants12051142)
Supplement: Supplementary file 1 [file plants-12-01142-s001.zip › plants-2223039-supplementary.pdf]

Supplementary Materials for

From the Wild to the Field: Documentation, Propagation, Pilot Cultivation, Fertilization, and Phytochemical Evaluation of the Neglected and Underutilized *Amelanchier ovalis* Medik. (Rosaceae)

Eleftherios Karapatzak [1,*,†], Giorgos Patakioutas [2], Katerina Papanastasi [1], Olga Dichala [1], Antonis Karydas [1], Nikos Nikisianis [3], Theodora Papagrigoriou [4,†], Diamanto Lazari [4], Nikos Krigas [1,*] and Eleni Maloupa [1]

[1] Institute of Plant Breeding and Genetic Resources, Hellenic Agricultural Organization Dimitra, 57001 Thessaloniki, Greece
[2] Department of Agriculture (UOI), School of Agriculture, University of Ioannina, 47100 Ioannina, Greece
[3] Systems of Forest and Environmental Development (SYSTADA), 8 Amasia, 55133 Thessaloniki, Greece
[4] Laboratory of Pharmacognosy, School of Pharmacy, Aristotle University of Thessaloniki, 54124 Thessaloniki, Greece
* Correspondence: ekarapatzak@gmail.com (E.K.); nikoskrigas@gmail.com (N.K.); Tel.: +30-2310471110 (N.K.)
† Authors contributing equally.

Supplementary Table S1. Overview of the conventional and organic fertilization regimes applied in the pilot field trial of *Amelanchier ovalis* subsp. *ovalis* GR-1-BBGK-04,2547 genotype during the experimental period (2020–2022).

**Crop Year**	**Fertilization Period**					
	**March**	**April**	**May**	**June**	**September**	**November**
			**Conventional fertilization**			
1rst year-2020	Peat (planting pit)	60 g/plant 21% N— 17% P_2O_5— 0.15% Zn—4% S	Fe: 20 g + Zn: 5 mL + B: 5 mL/10 L	60 g/plant N 13,7%, K2O 46,3%	-	35 g/10 L 46—0—0
2nd year-2021	180 g/plant 21%N- 17% P2O5- 0,15% Zn- 4%S	-	Fe: 50 g + Zn: 10 mL + B: 10 mL/10 L	180 g/plant 13,7% N, 46,3% K_2O	-	75 g/10 L 46—0—0
3rd year-2022	-	260 g/plant 21%N- 17% P2O5- 0,15% Zn- 4%S	Fe: 50 g + Zn: 10 mL + B: 10 mL/10 L	220 g/plant N 13,7%, K2O 46,3%	-	80 g/10 L 46—0—0
			**Organic fertilization**			
1rst year-2020	100 g zeolite + 50 g biocompost + 30 g P-30 + 30 g organic fertilizer (water-soluble Fe 12%w/w, water-soluble Mn 0.55% w/w, water-soluble Zn 0.49% w/w, total MgO 5.1% w/w, water-soluble MgO 3%w/w, water-soluble SO_3 37% w/w) (planting pit)	50 g natural product (2% organic acids, as complexes of natural aluminosilicate minerals and hydrated copper sulphate, adsorbed on the natural crystal)/10 L water	20 g organic fertilizer (organic water-soluble N 11% w/w, organic C 40% w/w, total amino acids 69.2% w/w) + 1.5 g organic fertilizer [organic & humic compounds: 68–78% (humic acids 40% min.), nutrient inorganic elements: 5% N, 3% P_2O_5, 3–5% CaO, 0.7–1.0% MgO, 1.2 Fe & trace elements (Zn, B, Cu) in ppm]+ 5 mL natural product (amino acids from vegetal organic matrixes, natural cytokinins, folic acid, humic and fulvic	20 mL organic biostimulator (5% w/w total aminoacids, 1.5% w/w free aminoacids, 10% w/w organic carbon, 10 mg/kg natural triacontanol)/10 L water	100 g natural product (2% organic acids, as complexes of natural aluminosilicate minerals and hydrated copper sulphate, adsorbed on the natural crystal 12.5% w/w)/10 L water	20 mL organic fertilizer (total N 2% w/w, organic N 1% w/w, water-soluble K2O 9% w/w)/10 L water

			acids, glutamic acid, asparagine, alanine, lysine, vitamins (A, B, C, PP, K), carbohydrates, micronutrients)/10 L water			
			50 g natural product (organic acids, organic calcium and boron sources and 3% of vitamins as complexes of natural aluminosilicate minerals) + 20 mL organic biostimulator (5% w/w total aminoacids, 1.5% w/w free aminoacids, 10% w/w organic carbon, 10 mg/kg natural triacontanol)/10 L water carbon, 10 mg/kg natural triacontanol)/10 L water			
2nd year-2021	50 g natural product (2% organic acids, as complexes of natural aluminosilicate minerals and hydrated copper sulphate, adsorbed on the natural crystal)+ 2 gr organic fertilizer	50 g natural product (2% organic acids, as complexes of natural aluminosilicate minerals and hydrated copper sulphate, adsorbed on the natural crystal)+ 5 gr Plant Growth Regulators–auxins, cytokinins,	20 g organic fertilizer (organic water-soluble N 11% w/w, organic C 40% w/w, total amino acids 69.2% w/w) + 1.5 g organic fertilizer [organic & humic compounds: 68–78% (humic acids 40% min.), nutrient inorganic elements: 5% N, 3% P_2O_5, 3–5% CaO,	20 mL organic biostimulator (5% w/w total aminoacids, 1.5% w/w free aminoacids, 10% w/w organic carbon, 10 mg/kg natural	100 g natural product (2% organic acids, as complexes of natural aluminosilicate minerals and hydrated copper	20 mL organic fertilizer (total N 2% w/w, organic N 1% w/w, water-soluble

	[organic & humic compounds: 68–78% (humic acids 40% min.), nutrient inorganic elements: 5% N, 3% P2O5, 3–5% CaO, 0.7–1.0% MgO, 1.2 Fe & trace elements (Zn, B, Cu) in ppm]/10 L water	gibberellins, betaine, polyamines > 300 ppm – polyphenols, polysaccharides, organic macronutrients (N,P,K,Ca,Mg,S) and trace elements (Mn,Cu,Fe,Zn,Mo,Co,B), fulvic acids (FA), amino acids and chelating agents (Mannitol), and in addition: Amino acids: 3-4%, Mannitol: 5-8%, Alginic acid: 14-16%, Organic matter: 40-50% /10 L water	0.7–1.0% MgO, 1.2 Fe & trace elements (Zn, B, Cu) in ppm]+ 5 mL natural product (amino acids from vegetal organic matrixes, natural cytokinins, folic acid, humic and fulvic acids, glutamic acid, asparagine, alanine, lysine, vitamins (A, B, C, PP, K), carbohydrates, micronutrients)/10 L water	triacontanol)/10 L water	sulphate, adsorbed on the natural crystal 12.5% w/w)/10 L water	K_2O 9% w/w)/10 L water
			50 g natural product (organic acids, organic calcium and boron sources and 3% of vitamins as complexes of natural aluminosilicate minerals) + 20 mL organic biostimulator (5% w/w total aminoacids, 1.5% w/w free aminoacids, 10% w/w organic carbon, 10 mg/kg natural triacontanol)/10 L water			
3rd year-2022		20 g organic fertilizer (organic water-soluble N 11% w/w, organic C 40%	50 g natural product (organic acids, organic calcium and boron sources and 3% of	20 mL organic biostimulator (5% w/w total	100 g natural product (2% organic acids,	20 mL organic fertilizer

	w/w, total amino acids 69.2% w/w) + 1.5 g organic fertilizer [organic & humic compounds: 68–78% (humic acids 40% min.), nutrient inorganic elements: 5% N, 3% P_2O_5, 3–5% CaO, 0.7–1.0% MgO, 1.2 Fe & trace elements (Zn, B, Cu) in ppm]+ 5 mL natural product (amino acids from vegetal organic matrixes, natural cytokinins, folic acid, humic and fulvic acids, glutamic acid, asparagine, alanine, lysine, vitamins (A, B, C, PP, K), carbohydrates, micronutrients)/10 L water	vitamins as complexes of natural aluminosilicate minerals) + 20 mL organic biostimulator (5% w/w total aminoacids, 1.5% w/w free aminoacids, 10% w/w organic carbon, 10 mg/kg natural triacontanol)/10 L water	aminoacids, 1.5% w/w free aminoacids, 10% w/w organic carbon, 10 mg/kg natural triacontanol)/10 L water	as complexes of natural aluminosilicate minerals and hydrated copper sulphate, adsorbed on the natural crystal 12.5% w/w)/10 L water	(total N 2% w/w, organic N 1% w/w, water-soluble K_2O 9% w/w)/10 L water

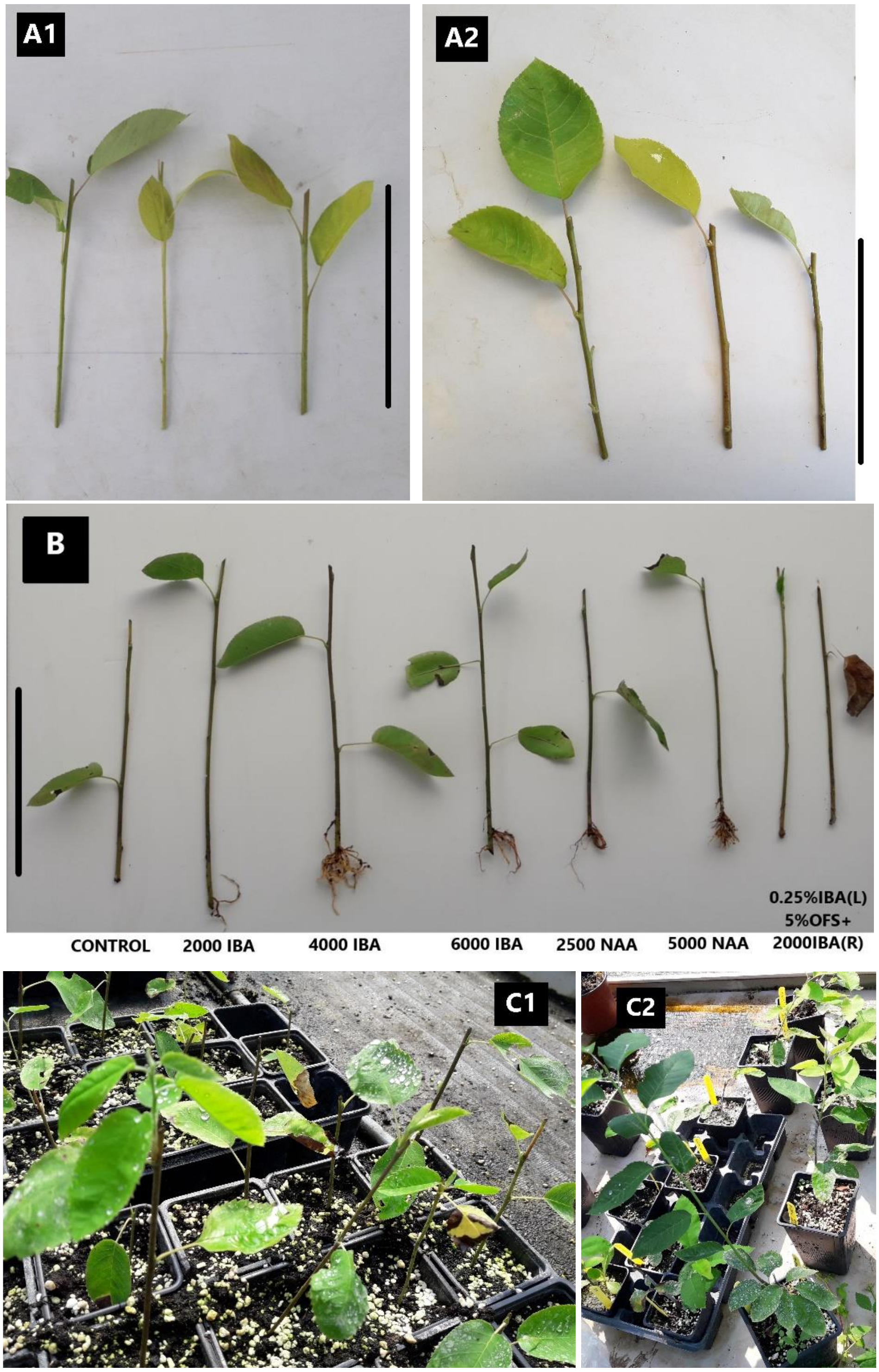

Supplementary Figure S1. Indicative photos of cutting propagation of the Greek native genotype of *Amelanchier ovalis* subsp. *ovalis* GR-1-BBGK-04,2547. (**A1**) Prepared soft-wood cuttings (experiment 1); (**A2**) Prepared cuttings for the second experiment; (**B**) Rooting results (experiment 1) across eight of the 13 applied treatments, i.e., Control, 2000 ppm indole-3-butyric acid (IBA), 4000 ppm IBA, 6000 ppm IBA, 2500 ppm 1-Naphthaleneacetic acid (NAA), 5000 ppm NAA and the failed treatments 0.25% powder IBA and 2,000 ppm IBA pre-treated with 5% organic fertilizer solution (OFS); (**C1**): Rooted individuals; (**C2**): Acclimatized *ex situ* raised plants. Bars in photos represent 10 cm.

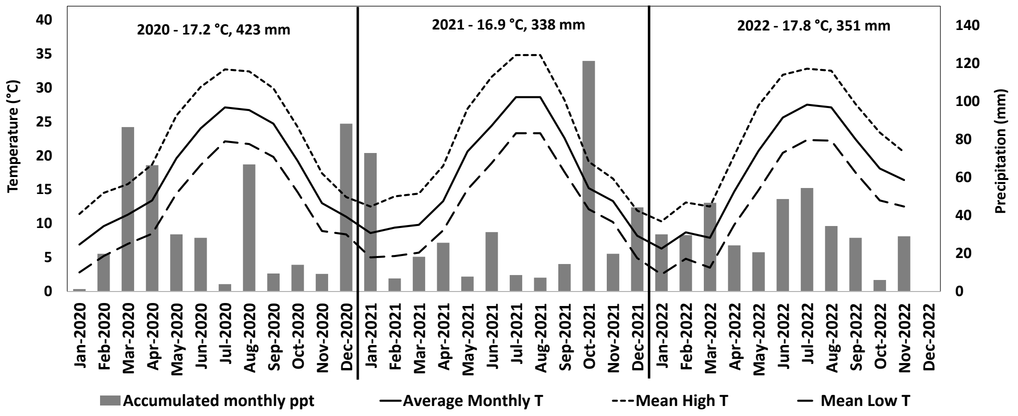

Supplementary Figure S2. Temperature and rainfall variation during the experimental period (2020, 2021, 2022; with average temperature and the total precipitation for each year) of the pilot field trial study in the experimental grounds of Thermi, Thessaloniki, Greece (elevation 40 m) presented yearly as monthly average temperature (°C, T) patterns coupled with mean monthly high and mean monthly low temperatures (°C, T) and accumulated total monthly precipitation (ppt, in mm) patterns.
